# Common Variable Immunodeficiency Disorders: A perspective from New Zealand

**DOI:** 10.1007/s12016-025-09099-2

**Published:** 2025-12-01

**Authors:** Rohan Ameratunga, Hilary J. Longhurst, Klaus Lehnert, Euphemia Leung, Richard Steele, See-Tarn Woon

**Affiliations:** 1 Department of Clinical Immunology, Auckland Hospital, Park Rd, Grafton, 1010 Auckland, New Zealand; 2 Department of Virology and Immunology, Auckland Hospital, Park Rd, Grafton, 1010 Auckland, New Zealand; 3 Department of Molecular Medicine and Pathology, School of Medicine, Faculty of Medical and Health Sciences, University of Auckland, Auckland, New Zealand; 4 Department of Medicine, School of Medicine, Faculty of Medical and Health Sciences, University of Auckland, Auckland, New Zealand; 5 Maurice Wilkins Centre, University of Auckland, Symonds St, Auckland, New Zealand; 6 Applied Translational Genetics, School of Biological Sciences, University of Auckland, Auckland, New Zealand; 7 Auckland Cancer Society Research Centre, Faculty of Medical and Health Sciences, University of Auckland, Auckland, New Zealand; 8 Department of Respiratory Medicine, Wellington Hospital, Wellington, New Zealand

**Keywords:** Common Variable Immunodeficiency Disorders, Hypogammaglobulinemia, SCIG/IVIG, Vaccine Challenge Responses, Transient Hypogammaglobulinemia of Infancy, Epistasis

## Abstract

Common Variable Immunodeficiency Disorders (CVID) are the most frequent symptomatic Primary Immunodeficiency (PID) in adults and children. Patients with CVID present with predominant antibody deficiency with varying degrees of impaired cellular immunity. CVID was previously a diagnosis of exclusion, which led to considerable uncertainty about which patients would qualify for subcutaneous or intravenous immunoglobulin (SCIG/IVIG) replacement. Over the last twelve years, several sets of diagnostic criteria have been published which identify these disorders with greater precision. These new CVID diagnostic criteria assist with decisions on treatment, particularly SCIG/IVIG replacement. With the advent of massively parallel genome sequencing technologies, it has become apparent that a significant proportion of individuals with a CVID phenotype have an underlying causative genetic defect. If such a pathogenic variant is identified, these individuals are removed from the overarching diagnosis of CVID and are deemed to have a CVID-like disorder caused by a specific Inborn Error of Immunity (IEI). New Zealand has had a long-standing customized PID genetic testing program. Two novel autosomal dominant pathogenic variants causing CVID-like disorders, consequent to haploinsufficiency of Nuclear Factor kappa-light-chain-enhancer of activated B cells (*NFKB1*) and Transcription Factor 3 (*TCF3*), were identified in New Zealand families. The latter pathogenic variant was shown to have an epistatic interaction with *TNFRSF13B* (TACI) in a patient with a digenic CVID-like disorder. Epistasis is the synergistic, non-linear interaction between two or more genetic loci, leading to much more severe (or much milder) disease. This perspective reviews the current understanding of these disorders with contributions from three New Zealand-based studies: The Prospective NZ CVID and the NZ hypogammaglobulinemia sub-studies as well as a large retrospective case series of Transient Hypogammaglobulinemia of Infancy (THI). These clinical and genomic studies have offered insights into the complexities of these rare PIDs. This review examines current areas of uncertainty in the diagnosis of of these disorders.

## Introduction

The understanding of Common Variable Immunodeficiency Disorders (CVID) continues to evolve. CVID is the most frequent symptomatic Primary Immunodeficiency Disorder (PID) in adults and children [1, 2]. These disorders, which were previously diagnoses of exclusion, can now be identified with greater precision [3]. This perspective overviews the current understanding of CVID, including contributions from studies undertaken in New Zealand (NZ).

In 2005 two long-term nation-wide cohorts of primary antibody deficiency were established in NZ; the New Zealand CVID (NZCS) and Hypogammaglobulinemia (NZHS) sub-studies as well as a retrospective analysis of Transient Hypogammaglobulinemia of Infancy (THI) [4–6]. New Zealand has also had a long-standing customised PID diagnostic genetic testing program, which has led to the discovery of two new genetic defects, autosomal dominant pathogenic variants of Nuclear Factor kappa-light-chain-enhancer of activated B cells (*NFKB1*) and Transcription Factor 3 (*TCF3*), consequent to haploinsufficiency [7–9].

In this perspective, these antibody deficiency cohort studies are briefly described, followed by an overview of diagnostic criteria for CVID including current areas of uncertainty. The complex genetics of CVID and CVID-like disorders will also be addressed including the evolving role of risk alleles such as *TNFRSF13B* (TACI). Finally, the benefits (and some disadvantages) of genetic testing will be discussed and referral criteria are suggested for colleagues in other specialities. Where pertinent, illustrative case reports from New Zealand patients have also been included in this article.

### Description of Predominant Antibody Deficiency Cohort Studies from New Zealand

In 2005, adult patients with predominant primary antibody deficiency were invited to join the NZCS and NZHS, two long-term prospective, cohort studies. Both sub-studies were approved by the NZ Ministry of Health (MEC 06/134) and the Auckland Hospital Ethics Committees (3435).

Patients in the NZCS were diagnosed with CVID at the time of enrolment or commenced SCIG/IVIG for their PID within six months of enrolment [6]. The NZCS has enrolled approximately 70% of patients known to have CVID in New Zealand [10].

Patients in the NZHS had reduced IgG levels (< 7 g/l) but were not accepted for SCIG/IVIG replacement within six months of diagnosis. Seven patients in the NZHS later deteriorated and were treated with SCIG/IVIG [5]. Eight patients in the NZCS who spontaneously increased their IgG, discontinued SCIG/IVIG and were reassigned to the NZHS [11]. Because of changes in SCIG/IVIG treatment status over time, the numbers of patients have varied in publications from these studies. The reclassification of patients, based on current SCIG/IVIG treatment status, is likely to have enhanced the accuracy of these analyses [11].

Once patients were enrolled, detailed clinical history was obtained by an interviewer-assisted questionnaire. Laboratory results were also reviewed. New Zealand citizens and residents are assigned a unique National Health Index (NHI) number which electronically links case notes, laboratory tests and treatment, including subcutaneous or intravenous immunoglobulin (SCIG/IVIG) replacement. The NHI maintains updated patient contact information, which together with access to comprehensive government-funded healthcare, leads to high follow-up rates in New Zealand-based cohort studies.

### Evolution of Diagnostic Criteria for CVID

This section examines how diagnostic criteria have influenced the understanding of CVID over the last three decades. Diagnostic criteria can be very helpful where the cause(s) of a disorder is unknown. The original European Society for Immunodeficiency (ESID) and the Pan American Group for Immunodeficiency (PAGID) CVID criteria (1999) specified an IgG 2 SD below the mean with impaired vaccine challenge responses in a patient over two years of age [12]. CVID was a diagnosis of exclusion by the ESID/PAGID (1999) criteria. These ESID/PAGID (1999) criteria were formulated to allow a diagnosis of CVID in low resource settings such as developing countries. These diagnostic criteria were however relatively non-specific leading to uncertainty about which patients would qualify for SCIG/IVIG treatment [13].

**Fig. 1:**
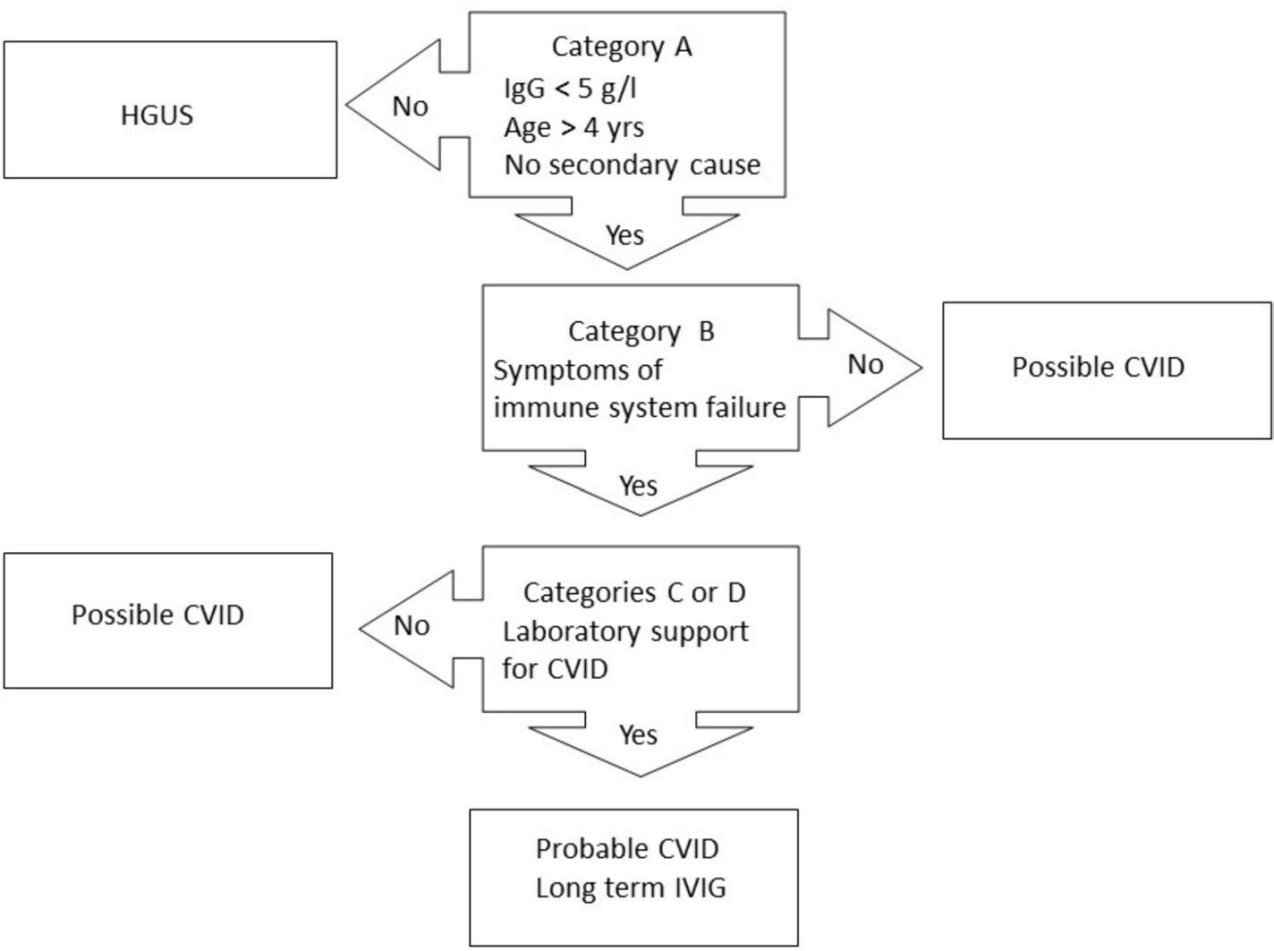
Diagnostic algorithm for SCIG/IVIG treatment. Patients who fulfil the criteria for CVID qualify for SCIG/IVIG. Asymptomatic patients with profound hypogammaglobulinemia should be treated with SCIG/IVIG as they may be at risk of meningitis, pneumonia or sepsis. [14]

In 2013 new diagnostic criteria were formulated, which allowed CVID to be diagnosed with greater precision (Table 1 and Fig. 1) [14]. The Ameratunga et al. (2013) criteria for CVID required an IgG of 5 g/l or lower [15]. Patients had to be symptomatic and be over four years of age. Supporting laboratory data including a reduction in IgA and/or IgM, reduced switched memory B cells or impaired vaccine challenge responses were required. Variants of genes predisposing to, or enhancing disease severity (risk alleles, described below) were also part of the criteria.

**Table 1:** New diagnostic criteria for CVID (Ameratunga et al. 2013) [14] Patients must fulfil category A and B criteria. Patients must then have either three category C criteria or one category D criterion to fulfil the diagnosis of probable CVID [30]

A	**Must meet all major criteria**
	• Hypogammaglobulinemia IgG < 5 g/l • No other cause identified for immune defect • Age > 4 years
B	**Sequelae directly attributable to immune system failure (ISF) (1 or more)**
	• Recurrent, severe or unusual infections • Poor response to antibiotics • Breakthrough infections in spite of prophylactic antibiotics • Infections in spite of appropriate vaccination e.g. HPV disease • Bronchiectasis and/or chronic sinus disease • Inflammatory disorders or autoimmunity
C	**Supportive laboratory evidence (3 or more criteria)**
	• Concomitant reduction or deficiency of IgA (< 0.8 g/l) and/or IgM (< 0.4 g/l) • Presence of B cells but reduced memory B cell subsets and/or increased CD21 low subsets by flow cytometry • IgG3 deficiency (< 0.2 g/l) • Impaired vaccine responses compared to age-matched controls • Transient vaccine responses compared with age-matched controls • Absent isohemagglutinins (if not blood group AB) • Serological evidence of significant autoimmunity e.g. Coombes test • Sequence variations of genes predisposing to CVID e.g. *TNFRSF13B*/TACI*, MSH5* etc
D	**Presence of relatively specific histological markers of CVID (not required for diagnosis but presence increases diagnostic certainty, in the context of Category A and B criteria)**
	• Lymphoid interstitial pneumonitis • Granulomatous disorder • Nodular regenerative hyperplasia of the liver • Nodular lymphoid hyperplasia of the gut • Absence of plasma cells on gut biopsy

Alternatively, CVID-defining histological lesions such as Nodular Regenerative Hyperplasia (NRH) of the liver could substitute for other laboratory abnormalities. Patients with profound asymptomatic hypogammaglobulinemia were deemed to have possible CVID. Patients with moderate reductions in IgG were considered to have hypogammaglobulinemia of uncertain significance (HGUS). These patients could either be symptomatic (sHGUS) or asymptomatic (aHGUS). These criteria were formulated to facilitate SCIG/IVIG treatment (Fig. 1). The Ameratunga et al. (2013) criteria have assisted in identifying several New Zealand patients with rare forms of secondary humoral immunodeficiency, who were initially thought to have CVID [16–18].

The following year the ESID registry criteria for CVID were published [19]. These were similar to the Ameratunga et al. (2013) criteria, with some differences. The reduction in IgG was set at 2 SD below the mean and there was a mandatory requirement for a reduction in IgA. Either impaired vaccine challenge responses or reduced switched memory B cells were needed. Asymptomatic patients with hypogammaglobulinemia could meet the ESID registry (2014) CVID criteria if they had an affected relative. Genes predisposing to or aggravating disease severity of CVID were not included in the ESID registry (2014) criteria. Similarly, there was no explicit comment on inclusion of the characteristic histological features of CVID in the diagnosis. Because of the uncertainties described here, the Ameratunga et al. (2013) and ESID registry (2014) criteria only allow a probable diagnosis of CVID.

The International consensus (ICON) criteria for a definitive diagnosis of CVID were published in 2016 [20]. The ICON (2016) criteria again specified an IgG below 2 SD with a reduction of IgA and/or IgM. Like the ESID/PAGID (1999) criteria, the ICON (2016) CVID criteria required impaired vaccine challenge responses. The ESID registry (2014) and the ICON (2016) criteria have set vaccine challenge responses at protective levels (tetanus and diphtheria toxoids at 0.1 IU/ml and H. influenzae at 0.15 µg/ml). In contrast, the Ameratunga et al. (2013) criteria have higher vaccine challenge thresholds, based on the responses of healthy adults (diphtheria and tetanus toxoids 1.0 IU/ml and HIB 1.0 µg/ml) [21, 22].

These diagnostic criteria have been compared in several studies [23–27]. Each has strengths and limitations [23]. In some cases, patients with severe primary humoral immune defects may not meet these criteria. As indicated previously, IgG levels and symptoms attributed to the primary hypogammaglobulinemia should be the most important considerations in making decisions on SCIG/IVIG treatment [14]. These patients should be managed by experienced adult or paediatric immunologists. In some cases, a consensus approach may be needed when making decisions on life-long SCIG/IVIG treatment [28].

## Areas of Uncertainty

### What IgG Threshold Should be Considered in the Diagnosis of CVID?

This section examines ongoing areas of uncertainty about the diagnosis of CVID. The ESID registry (2014) and ICON (2016) CVID criteria specify an IgG 2 SD below the mean [19, 20]. Although the population distribution of IgG does not follow a Gaussian curve, this is generally accepted as 7 g/l [29]. The NZHS showed that many patients with primary hypogammaglobulinemia can at least temporarily increase their IgG levels into the normal range [5]. This was termed Transient Hypogammaglobulinemia of Adulthood (THA). The explanation for THA is not known, as these patients did not have secondary disorders such as immunosuppression or malignancy. Seven of these patients with THA and one with delayed remission of THI were able to discontinue SCIG/IVIG treatment and have remained well [11].

THA has important implications for treatment with SCIG/IVIG, since asymptomatic patients with mild hypogammaglobulinemia may spontaneously normalize their IgG and might not require life-long SCIG/IVIG treatment. The ESID registry (2014) and ICON (2016) criteria require IgG levels to be repeated. Because of THA, the NZHS strongly supports the need for serial measurement of IgG levels, particularly if the IgG is > 6 g/l [6]. Like the French DEFI study [15], the IgG threshold was set at 5 g/l in the Ameratunga et al. (2013) criteria and the NZHS showed very few individuals normalised their IgG from levels below 5 g/l [5]. Future cohorts of patients with primary hypogammaglobulinemia will need IgG levels monitored over time and the IgG threshold for CVID diagnosis may need to be revised.

### Limitations in the Clinical Utility of Vaccine Challenge Responses in the Evaluation of CVID

Vaccine challenge responses play an important role in the diagnosis of CVID and in determining which patients should receive SCIG/IVIG [28]. It is assumed poor vaccine challenge responses reflect impaired in vivo humoral immunity. The ESID/PAGID (1999) and ICON (2016) criteria place paramount emphasis on impaired vaccine responses to establish a diagnosis of CVID, which would facilitate SCIG/IVIG treatment [12, 20].

In most cases of CVID, symptomatic antibody deficiency occurs after the primary childhood immunisation series. It is likely these patients have generated long-lived memory B cells, which could respond to future vaccine boosters. This was seen in the NZHS and NZCS, where almost all patients were able to mount protective antibody responses to tetanus toxoid and HIB [11]. Although there were statistically highly significant differences in vaccine challenge responses between patients accepted for SCIG/IVIG vs hypogammaglobulinemia patients who were not, there was considerable overlap in results. This compromises the predictive value of sub-protective vaccine antibody responses in the diagnosis of CVID. Like IgG levels, protein-based vaccine response thresholds may also need to be revised.

Responses to carbohydrate antigens are commonly assessed by antibody responses to vaccination with Pneumovax®. The American Academy of Asthma Allergy and Immunology (AAAAI) criteria are often used to determine appropriate Pneumovax® antibody responses in patients with hypogammaglobulinemia [30]. These require > 70% pneumococcal serotype responses over 1.3 µg/ml in adults and > 50% response in children. In the NZHS and NZCS vaccine response analysis, there were no statistically significant differences in Pneumovax®-induced antibody responses between patients accepted for SCIG/IVIG vs those who were not [31]. Criteria for appropriate Pneumovax® responses may also need to be revisited [32]. The situation will become even more complicated with the recent approval of Prevnar 20 for routine childhood immunisation [33]. The antibody response to S. typhi may be an alternative to Pneumovax® [34]. However, the S. typhi vaccine has not been validated in areas with a high prevalence of typhoid [35].

It may be more appropriate to examine responses to neoantigens in patients with late-onset antibody failure. Neoantigens such as the rabies vaccine or the φx 174 bacteriophage could be used in such patients [36]. There are however safety concerns about the routine use of the rabies vaccine and the φx 174 bacteriophage is not authorized for routine clinical use by the FDA [37].

### Variability of Memory B Cells Over Time

Most patients with CVID exhibit reduced numbers of switched memory B cells. This is likely to reflect impaired germinal centre function in these patients. Reduced switched memory B cells are part of the Ameratunga et al. (2013) and ESID registry (2014) criteria for CVID [14, 19].

There are three commonly used classification schemes for reduced memory B cells in CVID: The Freiberg, Paris and Euroclass criteria [38–40]. A New Zealand study examined the stability of these memory B cell subsets in patients presenting for IVIG each month [41]. It was apparent that there was considerable intraindividual variability in these monthly memory B cell tests. Consequently, the diagnostic category of many CVID patients changed during this six-month study. The variability was least in the Euroclass criteria, presumably because of the higher thresholds for switched memory B cells compared to the Freiberg and Paris classifications. This observation suggests memory B cells should be measured on more than occasion.

### The Critical Role of Histology in the Diagnosis of CVID

Histological features of CVID are underutilized in the diagnosis of CVID. CVID can be associated with several characteristic features including a granulomatous sarcoidosis-like variant (GVCVID), commonly affecting the liver, lymph nodes and lungs leading to Granulomatous-Lymphocytic Interstitial Lung Disease (GLILD) [42]. Other characteristic histological abnormalities of CVID include NRH of the liver and nodular lymphoid hyperplasia (NLH) of the intestines. Lymph node germinal centres from CVID patients frequently lack class-switched memory B cells and plasma cells.

These histological features are not entirely specific for CVID. However, in the context of primary hypogammaglobulinemia, they are CVID-defining lesions. These histological features can be very helpful in the diagnosis of CVID in patients, who may have a secondary cause. This was seen in a New Zealand patient with hypogammaglobulinemia who had GLILD, while on anticonvulsants [18]. Changing anticonvulsants resulted in partial recovery of immunoglobulins indicating she had underlying CVID, which was aggravated by concomitant anticonvulsant-induced hypogammaglobulinemia.

Histological features can be helpful in retrospective diagnosis of CVID, in patients being treated with SCIG/IVIG. Some of these patients have been on IVIG for decades and the original IgG levels may not be identified from case notes. Stopping SCIG/IVIG for several months could place these individuals at risk of severe bacterial infections including sepsis, meningitis and pneumonia. The relevant histological features may confirm the diagnosis without the need to stop SCIG/IVIG treatment.

### Overlapping Sub-Phenotypes of CVID Including Late-Onset Combined Immunodeficiency (LOCID)

Professor Helen Chapel was the first expert to demonstrate there are several overlapping clinical sub-phenotypes within the spectrum of CVID [43]. Some patients present with predominant gastrointestinal disease, while others have an infective phenotype with little evidence of inflammatory or autoimmune sequelae. It is important to note that the clinical features of CVID can evolve over time [44]. Young CVID patients with predominantly infectious complications can subsequently develop autoimmune disorders, especially autoimmune cytopenias. Inflammatory disorders such as NRH of the liver or NLH of the intestines can also occur later in the course of the disease, leading to death [45].

There is debate whether late-onset combined immunodeficiency (LOCID) should be considered a subset of CVID or a separate entity [46]. Some patients with severe T cell defects may benefit from Haematopoietic Stem Cell Transplantation (HSCT) and this has been an argument for separating these patients from those with CVID. Patients with LOCID can be identified during the clinical evaluation of patients with CVID, when relevant laboratory tests such as T cell subsets are undertaken.

The ESID registry (2014) and the ICON (2016) criteria exclude these patients from a diagnosis of CVID [19]. The ESID registry (2014) criteria specify T cell subset numbers in the exclusion of such patients. In contrast, the Ameratunga et al. (2013) criteria include patients with LOCID, providing a causative genetic variant has not been identified [46]. It is apparent there is a spectrum of cellular immunodeficiency in patients with CVID, which can evolve over time [1]. This is an argument for retaining patients with LOCID within the broad phenotypic spectrum of CVID.

### Delayed Remission of Transient Hypogammaglobulinemia of Infancy Complicates the Diagnosis of CVID in Children

**Fig. 2:**
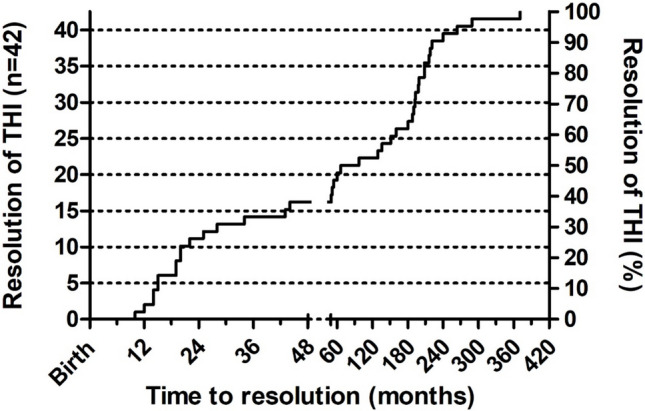
Kaplan–Meier plot of the NZ transient hypogammaglobulinemia of infancy (THI) study, showing the time course to recovery in 42 patients (with permission). [4] The x axis is in two segments, ages 0–4 years and 4–30 years. The majority of children did not remit until after 4 years of age, which will complicate the diagnosis of CVID in children, adolescents and young adults

The diagnosis of CVID remains an ongoing challenge, particularly in children. As noted in the NZCS and other cohorts, a large proportion of individuals later diagnosed with CVID in adulthood, date the onset of their symptoms to childhood. The three most recent CVID diagnostic criteria have raised the age threshold from two to four years of age because of the possibility of THI. The New Zealand THI study has however shown that most patients do not recover until after four years and many recovered in adolescence or even adulthood (Fig. 2) [4].

This poses a dilemma for paediatric immunologists. It is reasonable to undertake genetic studies in patients with primary hypogammaglobulinemia. If an IEI is identified, this excludes THI and obviates the need for diagnostic vaccine challenges, especially given their limitations, as discussed above [11].

If the child does not have an IEI following genetic testing and is symptomatic, he or she should be treated with prophylactic antibiotics and/or SCIG/IVIG. The diagnosis should be periodically reviewed. Where available, histological specimens should be carefully assessed. The presence of germinal centres with switched memory B cells supports a diagnosis of THI rather than CVID.

Memory B cells are typically reduced in CVID but some patients with THI can also have reduced memory B cells, which later recover [5]. It is reasonable to periodically measure switched memory B cells and IgA levels if these children are placed on SCIG/IVIG. Recovery of IgA levels and the generation of switched memory B cells suggests THI, rather than CVID.

When it is deemed safe to do so, SCIG/IVIG can be stopped in the summer months to determine if the IgG returns to the normal range. Accepting the limitations of the vaccine challenge responses, these studies could also be undertaken after stopping SCIG/IVIG [11].

### Presentation of Primary Antibody Deficiencies Later in Life

The prevalence of CVID in adults is at least 1:25 000 in Europe and America [25]. As noted, cohorts of CVID patients including the NZCS have shown that symptoms frequently begin in childhood although the diagnosis is often delayed until adulthood [2, 6, 47]. The NZCS showed that bronchiectasis and premature death are likely consequences of this diagnostic delay.

CVID studies have also shown onset of symptomatic disease later in life, sometimes in the seventh or eighth decades of life, in a minority [1]. Late-onset disease was also confirmed in the NZCS. A previous study and a more recent meta-analysis suggested a bimodal peak of CVID disease onset but the NZCS did not demonstrate this, probably because of smaller numbers of participants in the NZCS [1, 44].

### The natural history of untreated hypogammaglobulinemia in adults

Patients presenting with moderate primary hypogammaglobulinemia are a common scenario in clinical immunology practice. The NZHS demonstrated symptomatic state was a critical factor in long-term prognosis.

Patients who were minimally symptomatic had a good prognosis, remaining well for well over a decade. In contrast patients with similar levels of IgG who were symptomatic had a guarded prognosis. Many of these patients deteriorated clinically with a concomitant reduction in their IgG levels. Several such patients later met the criteria for CVID and were placed on long-term SCIG/IVIG treatment.

### Increased Rates of CVID in the Indigenous Māori of Aotearoa (New Zealand)

Current data indicates important ethnic differences in the prevalence of CVID [48]. Most case series have shown a predominance of CVID in Caucasians. In spite of large numbers of citizens of African, Chinese or South Asian ancestry, CVID cohorts from America, Australia and Europe comprise mostly Caucasian patients [1, 15]. CVID case series and databases from developed East Asian nations such as Japan, Singapore, South Korea and Taiwan confirm the lower numbers of CVID patients in these non-Caucasian populations [49, 50].

A recent study showed a surprisingly high prevalence of CVID in the indigenous Māori of Aotearoa (New Zealand) [6]. It is important to distinguish this observation from the high rates of autosomal recessive Inborn Errors of Immunity (IEIs) in consanguineous societies. Māori have a high rate of CVID compared with other non-Caucasian populations. The explanation for this observation is unclear as there does not appear to be a founder mutation causing an IEI in Māori. This observation could offer important insights into the immune pathophysiology underlying CVID. Future studies could compare differential expression of immune-related genes in different ethnic groups to determine the genetic basis of CVID.

### Disease Severity of CVID and CVID-Like Disorders

CVID and CVID-like disorders are associated with target organ damage. The lungs in particular are vulnerable to chronic suppuration leading to bronchiectasis. As noted in the NZCS, bronchiectasis is associated with a poor prognosis and premature death [6]. Other inflammatory sequelae including CVID-associated enteropathy caused by NLH of the intestines and NRH of the liver are also associated with poor outcomes [45].

The CDSS (Ameratunga score) was formulated to assess disease severity in these patients (Table 2) [51]. The CDSS is a cephalo-caudal review of CVID disease sequelae. Life-threatening complications are given a score of 10, while life-altering sequelae are scored with 5. Readily reversible complications are given a score of 1. Future studies will indicate if this approach will improve the management of these patients [52, 53].

**Table 2:** The CVID Disease Severity Score (CDSS; Ameratunga score) is an instrument for assessing CVID disease severity [51]. Life-threatening complications score 10, life-changing sequelae score 5 and readily reversible complication are given a score of 1. The total score can be computed from each organ system. The right-hand columns allow chronological documentation of cumulative disease burden. Two or more pneumonias within a five-year period constitute recurrent pneumonias. Similarly, two or more acute sinusitis or otitis media per year constitute recurrent disease

Parameter	Mild = 1	Moderate = 5	Severe = 10	Score/date
Malignancy			Present	
Lung	Mild asthma	Mild GLILD, mild bronchiectasis, moderate-severe asthma, uncomplicated pneumonia	Severe pulmonary dysfunction: Extensive bronchiectasis, complicated pneumonias, recurrent pneumonias, PJP, interstitial lung disease, lung surgery. Pseudomonas	
ENT	Otitis media, acute sinusitis	Chronic sinusitis, recurrent otitis media, recurrent sinusitis	Complicated mastoiditis	
Gut	Oral ulceration, oral candidiasis, Giardia, responding to treatment	Mild IBD, cholecystitis, esophageal candidiasis, celiac disease	Severe IBD, severe enteritis, severe malabsorption, peritonitis, unresponsive norovirus infection,	
Liver		Mild NRH, autoimmune hepatitis/granulomatous hepatitis responding to treatment	NRH with cirrhosis and/or portal hypertension, Viral hepatitis. Liver transplantation	
Cutaneous	HVS1 cold sores, mild cellulitis	Vitiligo, Extensive VVC, uncomplicated shingles		
Vasculitis		Cutaneous	Systemic	
Cytopenias	Mild asymptomatic	Requiring treatment	Poorly responsive cytopenias	
Spleen	Asymptomatic splenomegaly	Splenectomy		
Lymph nodes Non-malignant	Mild lymphadenopathy	Extensive incl sarcoid-like granulomatous disorder		
CNS			Severe involvement e.g. cauda equina syndrome, meningitis, ECHOvirus encephalitis, Cryptococcal meningitis, CNS vasculitis, granulomatosis etc	
Ocular		Uveitis responding to treatment	Sight threatening disease, e.g. keratitis, retinopathy or retinal vasculitis	
Cardiac		Pericarditis	Coronary vasculitis, myocarditis	
Renal	Uncomplicated UTI’s		Chronic renal failure from e.g. renal vasculitis	
Musculoskeletal	Arthralgia, myalgias, mild osteopenia	Arthritis, myositis, severe osteoporosis	Osteomyelitis	
Endocrine		ACTH deficiency	T1D	
Other infections		Non-life threatening abscesses	Sepsis, life threatening abscesses e.g. CNS, EBV/CMV lymphoproliferative disease	
Other autoimmunity	Hashimoto’s, pernicious anemia	Alopecia	SLE, APLS	
Nutrition	Uncomplicated Vitamin deficiency		Severe malnutrition BMI < 18	
Allergies	Allergic rhinitis, mild eczema	Severe eczema, food allergies, Multiple antibiotic allergies		
Iatrogenic complications		Moderate organ damage	Life-threatening complications	
Sundry	Novel sequelae	Novel sequelae	Novel sequelae	

*APLS*-antiphospholipid syndrome; *BMI*-body mass index; *GLILD*-Granulomatous-Lymphocytic Interstitial Lung Disease; *IBD*-inflammatory bowel disease; *NRH*-nodular regenerative hyperplasia of the liver; *PJP*-Pneumocystis jirovecii pneumonitis; *SLE*-Systemic Lupus erythematosus; *TID*-type 1 diabetes; *UTIs*-urinary tract infections; *VVC*-vulvovaginal condylomatosis. The last row has been left intentionally blank for future descriptions of novel CVID sequalae

The CDSS also serves as a checklist when patients are first seen in clinics and may be of assistance to physicians training in clinical immunology. A patient’s progress can be monitored by serial assessments of the CDSS. A rapidly increasing CDSS may signify a poor prognosis. These severely affected individuals may require aggressive treatment for example early HSCT, particularly if there is an underlying genetic defect.

### Genetics of CVID and CVID-Like Disorders

**Fig. 3:**
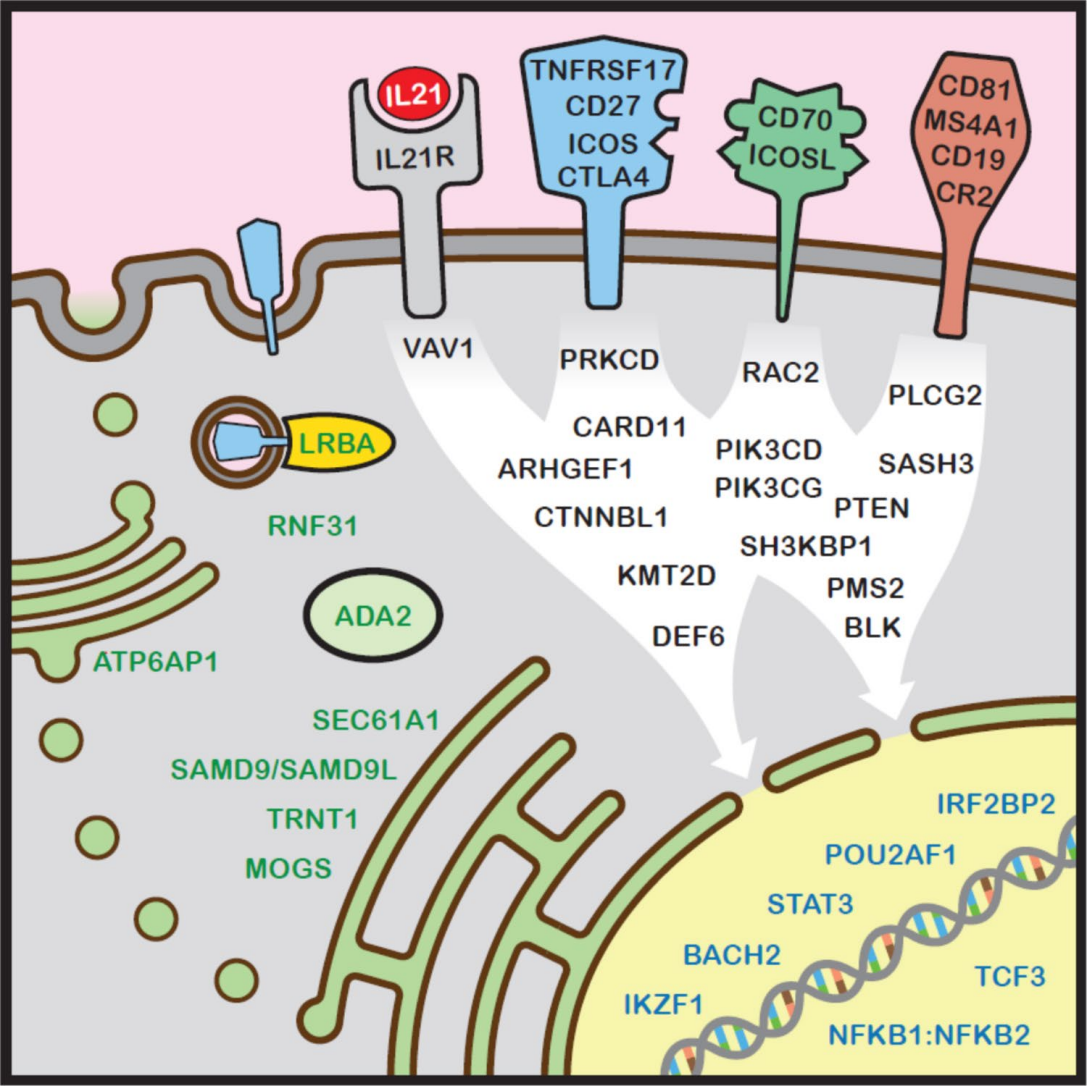
Some pathogenic gene variants associated with CVID-like disorders. The mechanism of action has been divided into cell surface molecules, intracellular signalling cascades and DNA-binding nuclear proteins. [54] Note that ADA2 may also have an extracellular role

This section examines the complex genetics underlying CVID and related disorders. This group of PIDs are an excellent example of locus heterogeneity (genocopy) [54]. Variants in seemingly unrelated genes can result in a broadly similar phenotype of predominant humoral immunodeficiency (Fig. 3). Until recently, identifying the genetic basis of disorders with marked locus heterogeneity has been challenging. Serial Sanger sequencing of multiple genes was not a sensible use of precious resources [55].

**Fig. 4:**
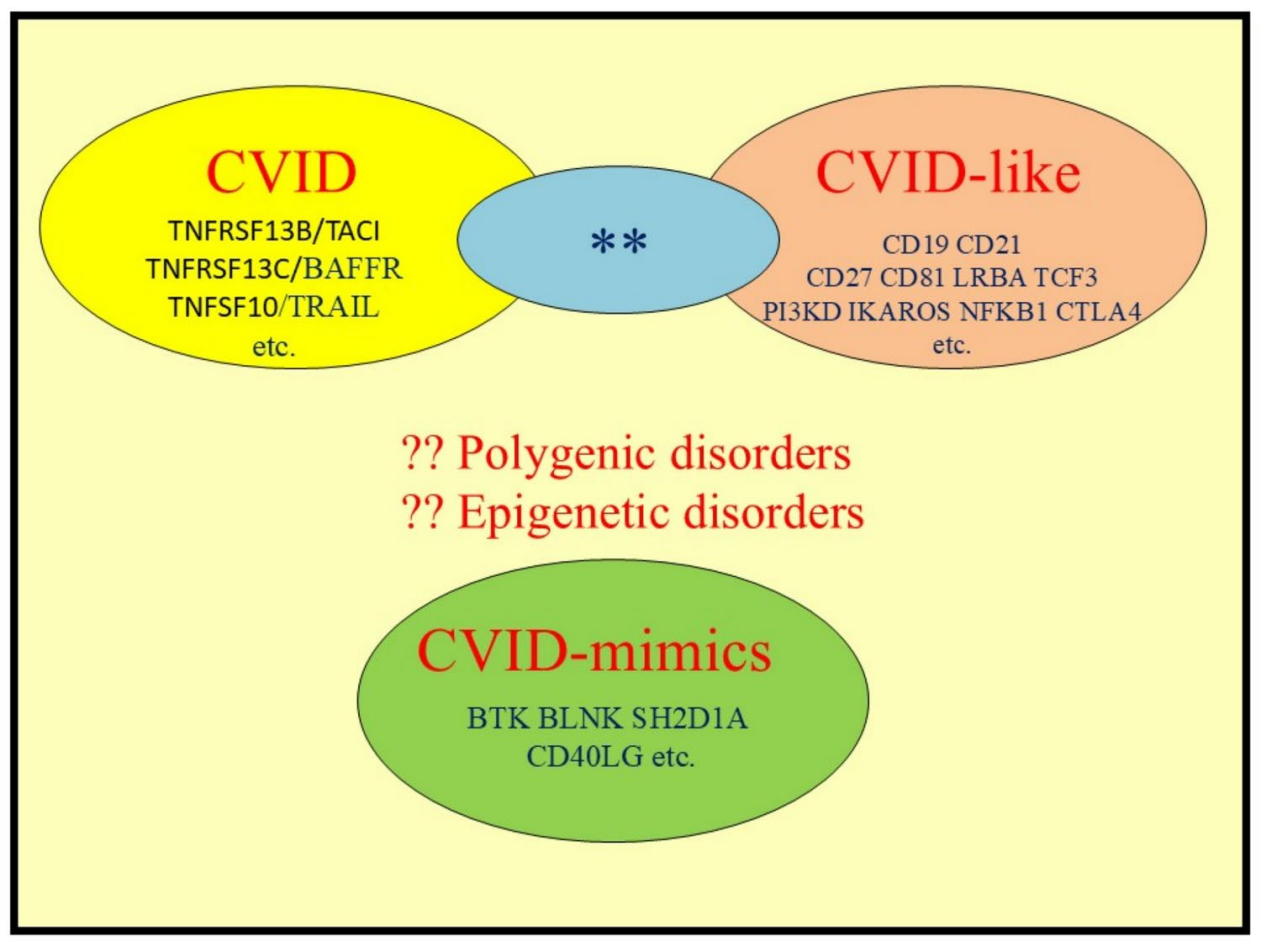
Genes associated with CVID, CVID-like disorders and CVID-mimics. CVID associated genes are those that are risk alleles and either predispose to CVID or enhance disease severity. ** Rarely specific variants usually associated with CVID disease predisposition or enhancement, can be causative. Current examples include *TNFRSF13B*/TACI (C104R) homozygous variants and the *TNFRSF13C*/BAFFR (P21R) variant. Genes causing CVID-like disorders will produce symptoms in the majority of individuals at some time in their lives. CVID mimics can occasionally present with predominant antibody deficiency but usually cause entirely different phenotypic features in the majority of affected individuals

With the advent of Massively Parallel Sequencing, which is the basis of Second Generation Sequencing (SGS), it has become feasible to explore the genetic basis of these disorders (Table 3) [56]. Studies from around the globe have shown that the genetics of CVID are complex (Fig. 4). As discussed below, all current CVID diagnostic criteria exclude patients with an underlying cause, which by definition, includes pathogenic variants [14, 19, 20]. If a causative genetic defect is identified, these individuals are deemed to have a CVID-like disorder caused by a specific IEI (Fig. 4) [31]. It should however be noted that the majority of patients with PIDS including those with selective IgA deficiency, THI and CVID do not have an IEI, as there is no known genetic defect [57]. Furthermore, it is debatable if a de novo pathogenic variant, yet to be passed to progeny can be considered an IEI.

**Table 3:** Limitations and potential errors of genetic testing from the perspective of the test cycle [56]. It is suggested these topics are discussed with the patient as part of informed consent for genetic testing

**Preanalytical**
• In most individuals with “CVID” from non-consanguineous societies, no deleterious variant (mutation) is identified
• Risk of genetic discrimination
• Disclosure of “incidentals” which are unrelated genetic mutations causing severe disease such as cancer or dementia (only for WES or WGS)
• Cultural issues may inform the protocol and location of genetic testing
**Analytical**
• Variable sequencing read coverage during WES- risk of missing a mutation
• Gene panels become obsolete with the discovery of new genes
• WES may miss complex variants such as gene rearrangements or copy number variations
• WES will miss intronic variants e.g. splicing errors or promoter pathogenic variants
• WES and WGS cannot identify variants in genes which have closely related pseudogenes with short read sequencing
• Sanger sequencing may be required before PGD and CVS
**Postanalytical**
• GoF variants can be difficult to identify by in silico methods
• Variants of Uncertain Significance (VUS) can lead to frustration in families, unless discussed prior to testing
• Functional studies may not be readily available
• Ethnic specific variants can lead to misdiagnosis
• In silico prediction tools can produce contradictory results
• Experimental trio analysis may lead to incorrect disease assignment with late onset disorders and may require re-analysis if a family member becomes symptomatic
• Non-paternity and occasionally non-maternity (e.g. ovum donation) can complicate analysis

*CVS*-Chorionic Villus Sampling; *GoF*-Gain of Function; *PGD*-Preimplantation Genetic Diagnosis

Autosomal recessive IEIs predominate with severe, early-onset disease in consanguineous societies [58]. In non-consanguineous families, late-onset autosomal dominant disorders account for a significant number of patients with primary antibody deficiency disorders [59]. Families with autosomal dominant CVID-like disorders demonstrate variable penetrance and expressivity [8, 60]. Some family members carrying these pathogenic variants become severely ill and succumb early, while others with the same ancestral mutation may remain well for the rest of their lives [46].

The explanation for variable penetrance and expressivity is not clear but possibilities include an environmental trigger such as COVID-19 or Herpes virus infections, which could alter their prognostic trajectory [61]. As discussed below, epistasis, which is the non-linear, synergistic interaction between two (or more) genetic loci, is another mechanism of variable penetrance and expressivity. Imprinting through altered gene methylation and expression is another recently described mechanism of variable penetrance and expressivity [60].

Although initially thought to be causative, it has become apparent that variants of some genes appear to predispose to, or enhance disease severity of CVID and are thus risk alleles, rather than causes of CVID-like disorders (Fig. 4). Variants in genes including *TNFRSF13B* (transmembrane activator calcium modulator cyclophilin ligand interactor; TACI), *MSH5* (Mut S homolog 5), *TNFSF10* (Tumour Necrosis Factor Related Apoptosis inducing Ligand; TRAIL), *TNFSF12* (TNF-like weak inducer of apoptosis; TWEAK) and *TNFRSF13C* (B cell activating factor receptor; BAFFR) seem to predispose to, or enhance disease severity in CVID [62].

The conclusion that these variants are risk alleles is based on several observations: The population prevalence of these variants far exceeds that of CVID making causality unlikely [62]. The American College of Medical genetics (ACMG) has published helpful guidelines on attributing causality to genetic variants [63]. As noted in Table 3, the ACMG criteria can be very helpful in resolving variants of unknown significance (VUS). These criteria have been applied to CVID and CVID-like disorders [64]. A much higher population prevalence of such variants than that of the disease (CVID), makes it less likely these are the cause of CVID-like disorders.

Family studies are another important consideration in the ACMG criteria for causality. Family studies have shown that these variants do not segregate with the phenotype (CVID) [65]. Discordant family segregation studies are further evidence against causality of these variants. It is important to include older relatives in such family studies as some of these disorders present later in life, consequent to variable penetrance and expressivity.

**Fig. 5:**
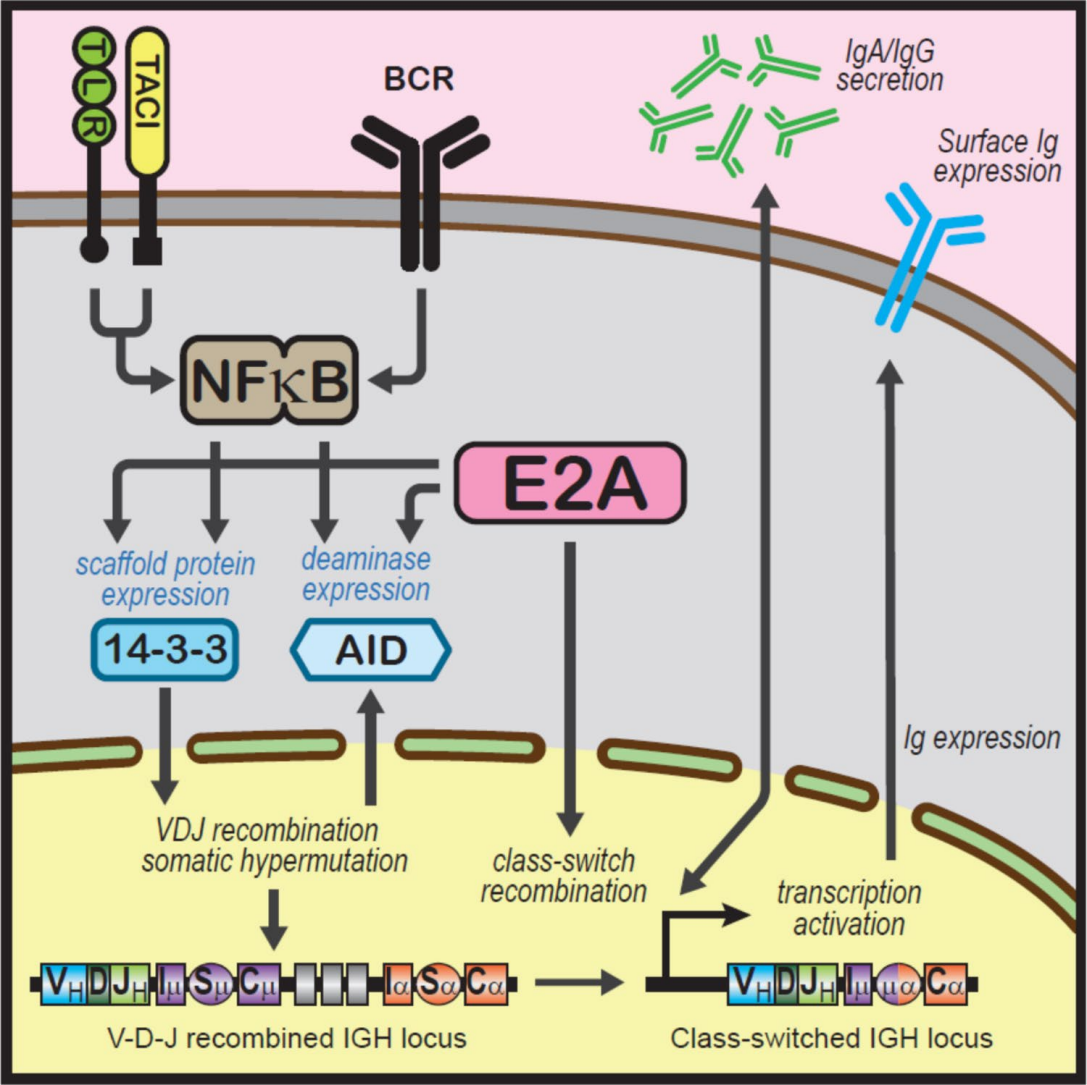
Epistatic interaction between variants of *TCF3* and *TNFRSF13B*/TACI. [66] *TNFRSF13B*/TACI plays a role in the alternative immunoglobulin production pathway. The E2A transcription factors, encoded by *TCF3* play a role in immunoglobulin class-switch recombination and production. The two genetic variants lie on the same pathway, leading to epistasis as seen clinically and from in vitro antibody production studies

Lastly, the epistatic role of these variants was directly demonstrated in a New Zealand family carrying a concomitant pathogenic variant of *TCF3* (Transcription Factor 3, Fig. 5) [66]. As noted, quantitative epistasis is the synergistic, non-linear interaction between two or more genetic loci leading to much more severe (or much milder) disease in comparison with individuals with a single genetic variant [67].

The proband who had both a missense *TNFRSF13B/*TACI (C104R, c.310T4C) variant and a nonsense variant of *TCF3* (T168fsX191) was much more severely affected than other family members bearing only one of these variants [66]. The proband had a higher clinical CVID Disease Severity Score (CDSS, Table 2) compared to other family members [51]. Laboratory studies mirrored the pattern of variants, where the digenic proband had the lowest in vitro antibody production, compared to other family members bearing only a single pathogenic variant.

There was thus congruence between in vivo and in vitro observations, confirming epistasis (Fig. 5) [67]. In this case, the *TCF3* nonsense variant was the epistatic hub, with a greater impact on the phenotype than the missense *TNFRSF13B/*TACI variant. With the routine use of SGS, it is likely many more patients with digenic and higher order polygenic defects will be identified in the future [68]. The current IUIS classification of monogenic IEIs will need to be revised, given the example above. It is important to note that not all polygenic disorders are examples to epistasis. Mutations in unrelated genes may have only an additive effect and epistasis might not occur [67]. An informative family carrying various permutations of the two (or more) variants is essential for the ascertainment of epistasis [67].

With the evolution of the understanding of these complex disorders, it has become apparent there may however be overlap between these sets of genes (Fig. 4) [69]. There are rare variants such as homozygous mutations of *TNFRSF13B*/TACI (C104R) or the recently described BAFFR (*TNFRSF13C*) (P21R) variant which could be considered causative [70].

Thus, in families with *TNFRSF13B*/TACI (C104R) variants some family members who are homozygous may have a CVID-like disorder, while other symptomatic heterozygous family members may have CVID (Fig. 4). It is likely other rare variants in these genes will be deemed to have a causative role, either in the heterozygous or homozygous state [69]. It will be important to curate each variant with the ACMG criteria to determine if it is a risk allele, or is a pathogenic variant underlying a CVID-like disorder. This section demonstrates the importance of routinely offering SGS to patients with a CVID phenotype and periodically repeating these analyses, given the rapid advances in knowledge of PIDs/IEIs [71].

Another area that remains to be elucidated is the role of epigenetics as a cause of CVID-like disorders. Monozygotic twins discordant for CVID were described a decade ago [72]. The authors provided evidence for methylation abnormalities in the affected twin. Genes including *TCF3* were impacted, which may have altered gene expression leading to a CVID-like disorder in the affected twin. It is possible future technologies will integrate methylation status into SGS. Since these changes are causative, CVID-like disorders may have an epigenetic basis in some cases.

Somatic pathogenic variants are recognised as having a causative role in some malignancies and are increasingly being documented in immunological disorders. Such somatic gene defects occur post-zygotically and are usually gain-of-function pathogenic variants leading to autoimmune or inflammatory disorders [73]. These have been classified as phenocopies by the IUIS expert committee on PIDS [31]. At this time, somatic variants have not been identified as a common cause of CVID-like disorders. They are however difficult to diagnose as the mean variant allele frequency may be low and detection may require enrichment of affected cells by techniques such as flow cytometry. By definition, somatic variants cannot be passed to progeny and it seems likely such variants might be important in sporadic, late-onset antibody deficiency disorders.

### The Clinical Utility of Diagnostic Genetic Tests in PIDs

The multiple overlapping benefits of PID genetic diagnosis have been previously described (Table 4) [74]. Identification of a causative genetic defect confirms the presence of a specific IEI. Elucidation of the relevant pathogenic variant can establish the diagnosis in atypical presentations. This is particularly important in autosomal dominant CVID-like disorders, which as noted, can have variable penetrance and expressivity [46].

**Table 4:** The many overlapping benefits of genetic diagnosis in PIDs including CVID-like disorders [80]. These should be discussed with patients prior to genetic testing along with potential disadvantages including genetic discrimination. Technical aspects of genetic testing shown in Table 3, should also be discussed as part of informed consent

**Securing the diagnosis**
• Atypical presentations e.g. autoimmunity, malignancy
• Distinguishing germline from somatic mutations
• Distinguishing one PID from another e.g. THI vs CVID-like disorders
• Distinguishing PID from secondary causes e.g. drug induced hypogammaglobulinemia
• The presence of a causative mutation obviates the need for vaccine challenges in CVID-like disorders
• Diagnosis where other methods are unavailable e.g. XLP1 and XLP2
• Diagnosis where a dysfunctional protein is expressed resulting in normal protein-based tests
• Identifying patients with digenic inheritance with or without epistasis
**Prognosis**
• Patients with a causative genetic defect will not recover spontaneously cf. THI
• Patients with causative genetic defects will need life-long monitoring and therapy
• Improved prognosis in patients undergoing HSCT for CVID-like disorders
**Therapy**
• Commencing SCIG/IVIG • Decisions about HSCT
• Use of specific drugs targeting the relevant mutation e.g. GoF of *PIK3CD*
• Gene based therapies including CRISPR Cas9
**Family Studies**
• Identifying asymptomatic individuals at risk
• Identifying atypical presentations consequent to variable penetrance and expressivity
• Carrier studies in female carriers of X-linked PID disorders at age of consent
**PID prevention**
• Preimplantation genetic diagnosis
• Chorionic villus sampling
**Research**
• Classification of PIDs. The IUIS does not currently have a category for polygenic PIDS/IEIS
• Identifying the role of defective molecules in disease pathogenesis
• Understanding the role of the same molecules in normal physiology
• Mapping out signaling pathways
• Exploring polygenic inheritance including epistasis
• Determining the epistatic hub in digenic inheritance
• Using this information for designer drugs e.g. mabs
• Determining the role of individual variants in polygenic disorders e.g. epistasis
• Generating animal models with the orthologous mutation by CRISPR Cas9

*GoF*-Gain of Function; *HSCT*-Hematopoietic stem cell transplantation; *Mab*-monoclonal antibodies; *THI*-Transient Hypogammaglobulinemia of Infancy; *XLP1*-X-linked lymphoproliferative syndrome type 1; caused by pathogenic variants of *SH2D1A*. XLP2- X-linked lymphoproliferative syndrome type 2 caused by pathogenic variants of *XIAP*

Genetic testing can identify many PIDs for which protein-based assays may be unavailable, such as *TCF3* haploinsufficiency [66]. Identification of the underlying genetic defect excludes other phenotypically similar conditions such as THI. Genetic testing can be helpful in determining prognosis, as in this example, where identification of a causative genetic defect excludes THI in a child. Unlike patients with THI, individuals with an IEI would not be expected to make a spontaneous recovery.

Other IEIs such as X-linked Hyper IgM syndrome (XHIM) and X-linked lymphoproliferative syndrome (XLP1) caused by pathogenic variants of *CD40LG* or *SH2D1A*, can occasionally present with antibody deficiency [75]. These CVID-mimics are fundamentally different from CVID and CVID-like disorders and are removed from further consideration as soon as the relevant genetic variants are identified (Fig. 4). Patients with these disorders may require specific treatment and some are amenable to curative HSCT.

Genetic testing can be helpful in family studies. Some individuals carrying the same mutation may be asymptomatic but may develop severe disease following an environmental trigger such as a viral infection. Asymptomatic carriers of autosomal dominant variants are at risk of transmitting the disorder to their progeny [46].

After appropriate counselling, genetic testing allows chorion villus sampling (CVS) and preimplantation genetic diagnosis (PGD). The New Zealand Government funds PGD including in vitro fertilization and embryo biopsy for patients with severe genetic disorders. This can interrupt transmission of the IEI to future generations. CVS and PGD require prior identification of the pathogenic mutation in one or both parents. It is not appropriate to offer CVS or PGD in most cases where genes predisposing to CVID have been identified, except in rare cases such as homozygous *TNFRSF13B*/TACI (C104R) variants, which could lead to a CVID-like disorder. Understanding the genetic complexities of CVID and CVID-like disorders is a prerequisite for genetic counselling and providing advice on CVS or PGD.

Identification of CVID-like disorders facilitates decisions on immunoglobulin replacement. SCIG/IVIG treatment should be based on symptomatic state and IgG levels in CVID-like disorders. Because CVID-like patients with IEIs are analogous to those with Bruton’s agammaglobulinemia, vaccine challenges are not a prerequisite to decisions on SCIG/IVIG replacement. This is a critical argument for SGS testing in patients after pretest counselling (Table 4).

Genetic testing may allow targeted treatment based on the variant. This may result in effective control of the disease with a lower risk of adverse effects. Examples include leniolisib or sirolimus for the Activated PIK3 Delta Syndrome (APDS) caused by pathogenic variants of *PIK3R* or *PIK3CD* genes or abatacept for CVID-like disorders caused by haploinsufficiency of *CTLA4 *[76]. These targeted therapies, in addition to SCIG/IVIG, are usually helpful when there is an autoimmune or inflammatory complication associated with these CVID-like disorders.

Identification of the underlying mutation is essential for gene-based therapies such as gene editing [77]. Finally, genetic testing can identify the physiological role(s) of these molecules in normal cellular function. IEIs have been termed experiments of nature [78].

Apart from clinical utility outlined above, there are substantial economic benefits of genetic testing, particularly for countries with socialised health systems such as NZ. Preventing a single case of a CVID-like disorder by PGD will save taxpayers NZD 2–3 million in lifetime costs of SCIG/IVIG alone. Gene panels currently cost less than NZD 1000 and preventing a single case of a CVID-like disorder would fund several thousand such tests.

While there are multiple advantages of genetic testing, limitations must also be discussed during pretest counselling (Tables 3 and 4). These include genetic discrimination in the domains of insurance and employment, in particular. Most countries, including New Zealand do not have enabling legislation such as the Americans with Disabilities Act (ADA, 1990) or the Genetic Information Non-Discrimination Act (GINA, 2009) [79].

### Viral Susceptivity in CVID

While most patients with a diagnosis of CVID have broad susceptibility to common bacterial pathogens, predisposition to viral pathogens is an area of investigation [81]. Patients with CVID may be at increased risk of severe COVID-19 caused by SARS-CoV2. Other common viral pathogens causing prolonged disease include norovirus. Whether patients with specific CVID-like disorders share the same susceptibility to different viral infections, remains to be determined [54]. It seems likely that large databases of such patients will provide helpful information on specific viral disease susceptibility in patients with CVID-like disorders in the future.

### Treatment of Non-Infectious Complications

Some autoimmune or inflammatory disorders require immunosuppression. This poses a dilemma, as these patients are already immunocompromised. They may be at risk of opportunistic infections following immunosuppression. Pretreatment work-up should comprise bone mineral density, Imaging, PCR studies of Hepatitis B and C and HIV. TB and other infectious diseases should be excluded. As part of the work up of such patients, immunisation against Shingles (Shingrix vaccine), influenza and SARS-CoV2 should also be considered, time permitting.

There may be geographic variations in the pre-immunosuppression workup, such as excluding endemic fungal disease in some parts of the world. Immunosuppression and its monitoring should be tailored to each case and these patients require close supervision. Literature should be reviewed and advice should be sought from Haematologists or Rheumatologists about the best treatment regimens for individual autoimmune or inflammatory disorders. Detailed treatment protocols for specific autoimmune and inflammatory sequelae are beyond the scope of this perspective.

### Observations on SCIG/IVIG Treatment from New Zealand

A study from New Zealand showed there is considerable risk in changing IVIG preparations. SCIG/IVIG preparations are not biologically equivalent. The New Zealand Blood Service is the sole supplier of SCIG/IVIG in NZ. When patients were changed from Intragam to Intragam P, there was a high rate of adverse reactions [82]. The conclusions from this study were that if there is a change in IVIG product, precautions such antihistamine and paracetamol prophylaxis should be undertaken, with a much slower initial infusion rate. Such a transition of SCIG/IVIG product must be planned well in advance and there should be full disclosure to physicians and patients prior to changing IVIG products.

There is ongoing debate about IVIG loading dose calculations in obese patients with CVID. A New Zealand patient undergoing bariatric surgery offered an opportunity to calculate IVIG dosage reduction [83]. The loading dose should be based on ideal body weight, while the maintenance dose of SCIG/IVIG should be based on the biological trough, where infective symptoms are mitigated.

### Clinical Features which May Alert Physicians to the Presence of CVID or other PIDS

PIDS can affect specific components of the immune system. Most predispose to infections, while others can present with predominant autoimmunity or malignancy. These patients can present to multiple specialties including Infectious disease, ORL, Respiratory medicine, Haematology/Oncology and Rheumatology with autoimmune disorders. Because of this marked phenotypic heterogeneity, it is important to maintain a high index of suspicion for the possibility of a PID. In a recent review, the following clinical features were suggested as indications for referring such patients to adult or paediatric immunologists (Table 5) [56].

**Table 5:** Clinical features which may indicate an underlying PID [56]. If there is suspicion of an underlying PID, it is suggested such cases are referred to an adult or paediatric immunologist, familiar with these disorders. SLE-Systemic Lupus Erythematosus

**Infectious History**
• Early onset of multiple infections
• Unusual sites of infections e.g. liver abscess • Unusual organism e.g. Pneumocystis jirovecii
• Unusual response to common infections e.g. Severe COVID-19 in a fully vaccinated child
• Poor response to antibiotics
• Other clinical features e.g. microthrombocytopenia in the context of recurrent or severe infections
• Chronic sinus disease
• Bronchiectasis
**Severe autoimmunity**
• Early-onset autoimmune disease
• Severe autoimmune disease- not responsive to usual treatments
• Multi-system autoimmune disorders e.g. cytopenias with autoimmune neurological disease
• Atypical laboratory findings e.g. ANA negative SLE when using HEP 2000 cells
**Malignancy**
• Lymphoid or solid neoplasia in the context of severe autoimmunity
**Family history**
• Family history of severe infections or unexplained deaths at an early age
• Family history of autoimmunity
• Family history of malignancy with autoimmunity
• Parental consanguinity increases the risk of autosomal recessive disease

## Conclusions

The understanding of CVID continues to advance. Apart from the indigenous Maori of Aotearoa, CVID (without a known causative genetic defect) is predominantly a disorder of Caucasians [84]. CVID can now be diagnosed with greater precision with the Ameratunga et al. (2013), the ESID (2014) or ICON (2016) criteria [14, 19, 20]. Each set of criteria has strengths and limitations, which have been compared in many studies around the world. In recent years it has become apparent that a large number of disorders resembling CVID have a causative genetic defect [54]. In some cases more than one gene may be affected [85]. Identifying the underlying IEI has profound implications for both the patient and their family. With the advent of SGS and progressive reduction in cost, patients with a CVID phenotype should be routinely offered genomic sequencing after pretest counselling [71].
